# Modern management of pyogenic hepatic abscess: a case series and review of the literature

**DOI:** 10.1186/1756-0500-4-80

**Published:** 2011-03-24

**Authors:** Helen M Heneghan, Nuala A Healy, Sean T Martin, Ronan S Ryan, Niamh Nolan, Oscar Traynor, Ronan Waldron

**Affiliations:** 1 Department of Surgery, Mayo General Hospital, Castlebar, Mayo, Ireland; 2 Department of Surgery, National University of Ireland, Galway, Ireland; 3 Department of Radiology, Mayo General Hospital, Castlebar, Mayo, Ireland; 4 Department of Histopathology, St.Vincent's University Hospital, Dublin, Ireland; 5 Liver Unit, St. Vincent's University Hospital, Dublin, Ireland

## Abstract

**Background:**

Pyogenic hepatic abscesses are relatively rare, though untreated are uniformly fatal. A recent paradigm shift in the management of liver abscesses, facilitated by advances in diagnostic and interventional radiology, has decreased mortality rates. The aim of this study was to review our experience in managing pyogenic liver abscess, review the literature in this field, and propose guidelines to aid in the current management of this complex disease.

**Methods:**

Demographic and clinical details of all patients admitted to a single institution with liver abscess over a 5 year period were reviewed. Clinical presentation, aetiology, diagnostic work-up, treatment, morbidity and mortality data were collated.

**Results:**

Over a 5 year period 11 patients presented to a single institution with pyogenic hepatic abscess (55% males, mean age 60.3 years). Common clinical features at presentation were non-specific constitutional symptoms and signs. Aetiology was predominantly gallstones (45%) or diverticular disease (27%). In addition to empiric antimicrobial therapy, all patients underwent radiologically guided percutaneous drainage of the liver abscess at diagnosis and only 2 patients required surgical intervention, including one 16-year old female who underwent hemi-hepatectomy for a complex and rare Actinomycotic abscess. There were no mortalities after minimum follow-up of one year.

**Conclusions:**

Pyogenic liver abscesses are uncommon, and mortality has decreased over the last two decades. Antimicrobial therapy and radiological intervention form the mainstay of modern treatment. Surgical intervention should be considered for patients with large, complex, septated or multiple abscesses, underlying disease or in whom percutaneous drainage has failed.

## Background

Pyogenic abscesses account for almost 80% of all liver abscesses in the developed world and are most often polymicrobial [1]. Amoebic and fungal abscesses are less common, and predominantly occur in Southeast Asia and Africa. Though relatively rare, with a reported incidence of 0.5-0.8% in the Western world and a frequency of 20 per 100,000 admissions in hospitalized patients, pyogenic hepatic abscesses are potentially lethal [2]. Hence there is a need to recognize and treat this condition urgently. The aetiology of these abscesses has changed over the last few decades. Historically, the commonest cause had been acute appendicitis but, with evolution and advancement of surgical practice and microbiology over time, its frequency as the primary source of abscess has decreased [3]. In contrast, the increasing frequency of cholelithiasis and biliary tract pathology, with their potential to incite ascending portal sepsis, have replaced appendicitis as the leading cause of hepatic abscess [2,4,5].

Liver abscess was previously regarded a high morbidity disease requiring open surgical drainage, with mortality rates between 9% and 80%. If untreated, it was uniformly fatal [4,6,7]. In the last quarter of a century we have witnessed a major paradigm shift in the management of pyogenic hepatic abscesses, with a concomitant decrease in mortality to 5-30% [8]. Advances in diagnostic and interventional radiology over the last three decades have facilitated a minimally invasive approach to management of this condition. In combination with targeted antimicrobial therapy, percutaneous drainage techniques now form the mainstay of treatment. However, a small proportion of patients do not respond appropriately to minimally invasive management strategies; it is critical to promptly recognize these patients, for whom traditional open surgical intervention is the definitive treatment. We have reviewed our experience in managing pyogenic liver abscess over the last 5 years, to illustrate the current etiology, management and outcomes of this disease. We also reviewed the literature in this field, and present a summary of current practice patterns which may serve as a useful guide for the modern management of pyogenic hepatic abscess.

## Methods

Our institution's ethical committee granted ethical approval for this study, which was then carried out in compliance with the Helsinki Declaration. Demographic and clinical details of all patients admitted to our unit with liver abscess(es), between June 2004 and June 2009, were prospectively entered onto a database. Data were retrieved from a combination of chart review, theatre records, Hospital In-Patient Enquiry (HIPE) and histopathology databases. A review of these data was performed to document the clinical presentation, aetiology, diagnostic work-up, treatment, morbidity and mortality. Radiologically-guided aspiration and drainage of abscesses was performed under local anaesthesia, with an 18-gauge needle and a range of different sized drainage catheters (8-12Fr) placed by the Seldinger technique. Pus aspirated at the time of initial abscess drainage was routinely sent for microbial culture and sensitivity analysis. Following completion of their treatment regimens, all patients were followed-up clinically and radiologically for at least 1 year to verify abscess resolution.

## Results

### Clinical presentation

Over a 5-year period, 11 patients presented to our unit with pyogenic liver abscess. Mean age was 60.27 years (range 16-85 years); there were 6 males and 5 females. Underlying comorbidities were present in7 of 11 patients (63.6%) including diabetes (n = 2), hypertension (n = 3), atrial fibrillation (n = 1), asthma requiring regular inhaled steroids (n = 1), and 2 patients had a history of diverticular disease (two patient had more than one comorbidity at presentation). No patient was taking immunosuppressant medications. The commonest presenting features were vague non-specific symptoms including fever (n = 7), right upper quadrant (RUQ) pain (n = 5), jaundice (n = 3), anorexia and malaise (n = 3). RUQ tenderness and pyrexia were present in the majority of patients (n = 8). Biochemical and haematological findings are documented in Table 1. The most consistent blood abnormality was an elevated C Reactive Protein (CRP), which was observed in all cases.

**Table 1:** Biochemical and haematological derangements in patients with liver abscess

Biochemical/haematological abnormality	% of cases (n)
↑ C-Reactive Protein	100% (11)
↑ White cell count	91% (10)
↑ Liver function tests	82% (9)
Normochromic normocytic anaemia	64% (7)
↑ Serum Creatinine	45% (5)

### Diagnostic work-up

**Figure 1:**
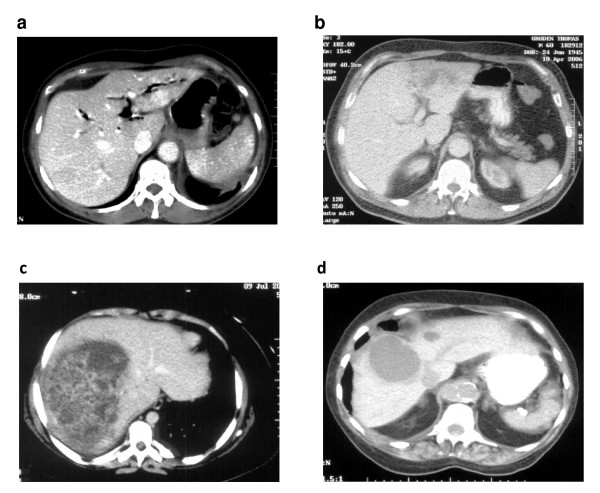
**(a-d) CT images of four patients with hepatic abscesses**.

All patients were initially evaluated with abdominal ultrasonography (US). Following confirmation of an abscess in the liver, computed tomography (CT) was performed to further define its anatomic features, and to try to determine its aetiology (Figure 1). The sensitivities of US and CT were 100% in this series. Abscess location was predominantly in the right lobe of the liver (72% of patients). Ten patients had a solitary abscess; only one patient in this series presented with multiple abscess cavities. All patients proceeded to undergo US-guided abscess drainage, either at the time of diagnosis or within 24 hours. Characteristics of these hepatic abscesses, with corresponding clinical data, are presented in Table 2.

**Table 2:** Characteristics of the liver abscesses

*Clinical presentation*					*Characteristics of hepatic abscess*				*Management details*	
**Case**	**Gender**	**Age**	**Predominant symptoms**	**Duration**	**Site (Lobe)**	**Number**	**Size (cm)**	**Likely etiology**	**Drain**	**Surgery**

1	Male	61	Fever, epigastric pain, anorexia/malaise	5 days	Right	Single	7	Portal vein sepsis*	Yes	Yes
2	Male	72	Fever, rigors	11 days	Left	Single	4	Portal vein sepsis*	Yes	No
3	Male	75	Fever, myalgia, pleuritic lower right chest pain	10 days	Right	Single	6	Portal vein sepsis*	Yes	No
4	Female	78	Fever, anorexia/malaise	2 weeks	Right	Single	10	Other^	Yes	No
5	Male	85	Anorexia/malaise	4 weeks	Right	Single	4	Other^	No	No
6	Female	56	RUQ pain, nausea	6 weeks	Right	Single	6	Biliary tract sepsis	Yes	No
7	Female	16	Fever, RUQ pain, jaundice	5 days	Right	Single	12	Actinomycosis	Yes	Yes
8	Female	53	RUQ pain	5 days	Left	Single	7	Biliary tract sepsis	Yes	No
9	Male	33	Fever, jaundice, dark urine	3 days	Right	Multiple	11	Biliary tract sepsis	Yes	No
10	Male	64	RUQ pain, jaundice	7 days	Right	Single	5	Biliary tract sepsis	Yes	No
11	Female	70	Fever, RUQ pain	5 days	Right	Single	10	Biliary tract sepsis	Yes	No

* Portal vein sepsis; secondary to gastrointestinal sources of infection, including diverticulitis and appendicitis

^ Other causes include: hematogenous seeding from a distant site of infection, or unknown etiology

### Aetiology

Gallstones were present on abdominal US in 5 patients (45.4%) although evidence of acute cholecystitis was only one present in one of these cases. One patient who presented with obstructive jaundice had evidence of intra-hepatic biliary dilatation on US, secondary to compression of the hepatic ductal confluence in the porta hepatitis by a left lobe abscess. CT scan identified diverticular disease in 3 additional cases (27.2%), without overt diverticulitis. In the remaining 2 cases there was no radiologically identifiable cause for liver abscess (18.2%).

### Microbiology

**Figure 2:**
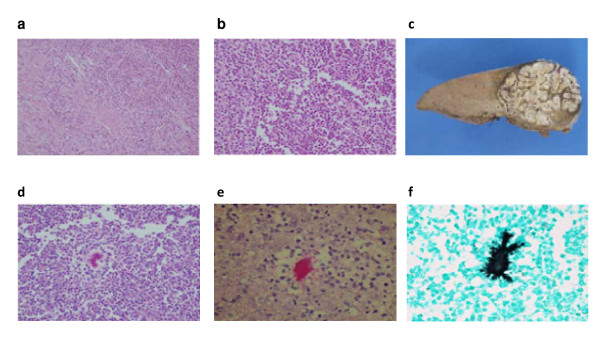
**Pathological images from two patients with hepatic abscess**: (a-b) Demonstrates pathology of a 61yr old male with a 7 cm abscess, of biliary aetiology, in the right lobe of his liver. (c-f) Gross and microscopic pathological mages from a 16-year-old Irish female who presented with a 12 cm Actinomycotic abscess in right lobe of liver. (2a) H/E section (x 200 mag) of a pyogenic liver abscess showing inflammatory cells. (2b) H/E section in higher magnification (x 400 mag) showing acute inflammatory cells forming an abscess. (2c) Section of liver illustrating a well circumscribed abscess cavity (2d) H/E section (x 200 mag) of a pyogenic liver abscess showing a microabscess with a central filamentous organism (Actinomycosis) (2e) PAS stain (x 400 mag) highlighting the actinomyces organisms (2f) Grocott stain (x 400 mag) highlighting the actinomyces organisms (black)

Microbial culture of pus drained from the hepatic abscess was positive in 7 cases (64%) whilst blood cultures were positive in only 2 cases. Gram positive cocci (Group B, Group C and Milleri Group Streptococci) were the most commonly isolated organisms. Coliforms were isolated in 3 cases (27%). Actinomyces was isolated from the abscess of a 16 year old Irish girl who had no history of immunocompromise, foreign travel, recent abdominal trauma or surgery. PAS and Grocott's stains demonstrated growth of the actinomyces organisms in clusters of tangled filaments surrounded by neutrophils (Figure 2).

### Treatment

All patients were empirically commenced on broad spectrum intravenous antibiotics at presentation. The most commonly used empiric antimicrobial regimen was a 3rd generation cephalosporin and metronidazole. This was changed according to the sensitivities of organisms cultured from each patient's abscess aspirate or blood. Ciprofloxacin was used as an alternative to a cephalosporin in one patient who had a history of penicillin anaphylaxis. All patients underwent radiologically guided percutaneous drainage of the liver abscess at the time of diagnosis or within 24 hours of presentation. In 10 cases (91%) a drain was placed in the abscess cavity and left in-situ following initial aspiration. Duration of drain placement ranged from 4 to 7 days. The timing of catheter removal was guided by repeat imaging (US) demonstrating a significant decrease in abscess size, or diminution of drain output along with improvement in clinical condition. One patient with a complex septated abscess required placement of a second drain after 36 hours, as his clinical condition did not improve and drain output was minimal. Four patients required transfer to the National Liver Unit as they failed to improve despite maximal non-operative primary therapy. Two of these patients (18% of all cases) underwent surgical intervention. A 61 yr old male, with a 7 cm complex septated abscess in the right lobe of his liver, underwent right hemi-hepatectomy due to progressive deterioration in his clinical condition despite percutaneous catheter drainage, and a suspicion of malignancy on preoperative imaging. The second operative case was that of a 16-year-old Irish female who presented with a two-week history of RUQ pain, fever, and anorexia. At presentation she had profound systemic sepsis. Abdominal US and CT demonstrated a large (12 cm) complex abscess in right lobe of liver and despite broad spectrum intravenous antibiotics, percutaneous aspiration and drainage, her clinical condition deteriorated and she underwent right hemi-hepatectomy and cholecystectomy for a complex Actinomycotic abscess.

### Outcome

There were no mortalities in this series. All patients made a full recovery, and demonstrated evidence of complete abscess resolution after a minimum of 1-year follow up. The overall morbidity rate was 45% (n = 5). Four patients developed severe sepsis with organ dysfunction over the course of their admission. These patients all required intensive care and transfer to a specialized hepatobiliary unit for further management. The young female with a primary Actinomycotic liver abscess required a 6-month course of oral penicillin post-operatively. One other patient developed a lower limb deep venous thrombosis (DVT) despite prophylactic treatment with low molecular weight heparin. Overall the mean length of hospital stay for patients managed non-operatively (n = 9) was 21 days. It was considerably longer for those patients who underwent surgical intervention (35 days, on average). All patients were followed up for minimum of 12 months following hospital discharge; repeat liver biochemistry and abdominal imaging (US or CT) confirmed abscess resolution in all cases.

## Discussion

Hepatic abscess was first described in the time of Hippocrates around 400B.C. In 1938, Ochsner's, seminal review of 47 cases of pyogenic liver abscesses heralded open surgical drainage as the definitive therapy. This series highlighted the uniformly fatal outcome of untreated abscesses. Despite the recommended aggressive approach to treatment, mortality rates throughout the mid twentieth century remained high at 60-80%. Advances in diagnostic and therapeutic radiology, coupled with improvements in microbiological identification and therapy, have recently decreased mortality rates to <5-30% [2]. In our series of 11 patients, there were no deaths and morbidity rate was 45%.

With the development of more sophisticated diagnostic techniques and longer lifespan, the demographic for peak incidence of liver abscess is shifting towards the sixth and seventh decades of life [9]. Liver abscesses are rare in children and adolescents, and are usually associated with an underlying immune deficiency state or trauma. Clinical presentation of pyogenic hepatic abscess is non-specific and similar to that of other hepatobiliary inflammatory or infectious process. A combination of nonspecific systemic symptoms is the most common presentation; including fever and rigors, nausea and vomiting, right upper quadrant pain, anorexia, weight loss, weakness and malaise. Less frequently reported symptoms include cough or hiccups due to diaphragmatic irritation, with referred pain to the right shoulder. Patients with small solitary lesions usually have a more insidious course with associated weight loss, fatigue and anaemia of chronic disease. With such symptoms, malignancy is often the initial concern. Multiple abscesses usually result in more acute presentations, as reflected by the one case in this series with multiple abscesses who presented after a short 3 day illness, with symptoms and signs of systemic toxicity [3,9]. Regarding laboratory abnormalities associated with hepatic abscesses, elevated inflammatory markers are sensitive but non-specific. In our series, we observed raised CRP in all cases. With regard to liver biochemistry, an elevated ALP is the single most common finding, observed in up to 90% of cases. Approximately 50% of patients demonstrate a general derangement of liver biochemistry, including elevated serum bilirubin, and hepatic enzymes AST and ALT. Other haematological abnormalities include leucocytosis and normochromic normocytic anaemia. Blood cultures are positive in less than 50% of cases [9]; we observed positive blood cultures in only 18% of our case series. Definitive diagnosis requires radiological evidence of an abscess in the liver parenchyma. Ultrasonography and computed tomography of the abdomen are the gold standard diagnostic modalities. In addition, they have therapeutic utility as they are employed to guide percutaneous aspiration and drainage of an abscess. These radiological investigations may also reveal the underlying aetiology, for example a contiguous infection within the biliary tree or peritoneal cavity.

Although the incidence of pyogenic liver abscess has remained relatively unchanged over time, the underlying aetiology has changed in unison with developments in surgical and microbiology practices. Biliary tract disease has replaced appendicitis as the commonest cause of bacterial liver abscess [4]. Chole(docho)lithiasis, obstructing tumours, strictures, and congenital anomalies of the biliary tree, are common causative conditions. An alternative mechanism for liver abscess formation, apart from direct spread from contiguous infection, is embolisation of a septic coagulum from an intra-abdominal infection. Diverticulitis, inflammatory bowel disease and perforated hollow viscera are possible sources of septic emboli in addition to appendicitis. Rarely, abscess formation results from haematogenous dissemination of organisms in association with a systemic bacteraemia, e.g. from endocarditis or pyelonephritis. Such cases have been reported in immunocompromised individuals with chronic granulomatous disease and leukemia [5]. Our series reflected this changing trend in aetiology; with a biliary source in 45%, diverticular aetiology in 27.5%, and likely haematogenous dissemination in the remaining 27.5% of patients. The organisms isolated from a liver abscess are varied, usually polymicrobial, and reflective of the infectious source (Table 3). Whilst this study and review focused primarily on bacterial causes of liver abscess, it should be noted that parasitic organisms are also common etiological factors, particularly in tropical and subtropical climates. *Entamoeba histolytica *and the nematode *Ascaris lumbricoides *are two such parasites associated with liver abscess formation, and should be considered in patients from endemic areas [1,10,11].

**Table 3:** Causative organisms of hepatic abscess

Source of infection	Common Organism
Biliary	Enteric gram negative organisms (enterococci)
Pelvic	Bacteroides fragilis
Other intraperitoneal source	Mixed aerobic/anaerobic organisms (e.g. B.fragilis)
Haematogenous seeding	Single organism usually e.g. Staphylococcus, Streptococcus (including Strep. milleri)
Immunocompromised	Candida species
Other	Pyogenic: Klebsiella pneumoniae (Asia), Actinomyces (rare). Amebic: Entamoeba histolytica (Amebic abscess),
	Parasitic: Ascaris lumbricoides

Untreated, pyogenic hepatic abscess is uniformly fatal due to ensuing complications such as sepsis, or peritonitis secondary to rupture of the abscess cavity into the pleural or peritoneal cavities. Systemic antimicrobial therapy remains the mainstay primary treatment. The choice of empiric antibiotics is based on the most likely source of infection, but should be broad spectrum and administered parenterally. The regimen must be reviewed and altered, if appropriate, to target specific organisms isolated from the abscess aspirate and/or blood cultures. The recommended duration of parenteral antibiotic therapy is 2-3 weeks, or until there is a favourable clinical response. Complementary oral antimicrobial therapy must then be continued for a further 2-4 weeks or until clinical, biochemical and radiological follow-up demonstrates complete resolution of the abscess cavity. Evidence suggests that antibiotic therapy alone is usually not sufficient to entirely resolve a liver abscess unless it is small (<3 cm) [12,13]. If antibiotics are selected as the sole therapeutic modality, then a prolonged course of systemic and oral therapy is warranted, and the choice of antimicrobial agent must be guided by the sensitivities of causative organisms [14,15]. There is debate however as to the size of abscess above which antibiotic treatment alone is unlikely to be successful. Malik *et al *recently reported their experience of managing 169 pyogenic liver abscesses, 16 of which were treated with intravenous antibiotics alone for 2 weeks [10]. This conservative approach was successful in only 6 of the 16 patients; the remaining 10 required open surgical drainage for definitive control of sepsis. The authors' criteria for choosing antibiotics as the single therapeutic modality were an uncomplicated abscess, <6 cm in size. The authors' low success rates with this approach provide support for draining abscesses smaller than 6 cm, as recommended by Krige *et al *[1]. Indeed various reports suggest that any abscess larger than 3 cm should be routinely drained [13,16,17]. In addition to abscess size, other criteria for percutaneous drainage include: continued pyrexia after 48-72 h of adequate medical treatment, and clinical or ultrasonographic features suggest impending perforation [10,18].

Surgical drainage is associated with high therapeutic success rates, and was the standard of care until the introduction of percutaneous drainage techniques in the mid 1970s. With refinement of image-guided techniques in recent years, percutaneous drainage and aspiration have emerged as appropriate alternatives to open drainage, providing similarly high success rates but with the advantages of a minimally invasive approach[19-21]. The decision to leave a drainage catheter in the abscess cavity following aspiration, rather than performing repeated needle aspirations, is also contentious. Those who prefer multiple aspirations believe it to be as effective and safe as catheter drainage, but simpler and quicker to perform, with decreased risk of procedural complications and post-procedural sepsis [17,22,23]. However, this approach requires careful follow-up, and often multiple repeat imaging procedures to monitor response to therapy. Insertion of a drainage catheter has been shown to be more effective than needle aspiration, particularly for abscesses larger than 5 cm. Failure rates are higher, and the average time to achieve a 50% reduction in size of an abscess cavity has been shown to be significantly longer, with needle aspiration compared to abscesses in which a drainage catheter has been placed [17,22].

Despite the reported success of percutaneous drainage techniques, there remains a role for open surgical intervention in the management of pyogenic abscess. Indications for surgical intervention include:

■ No clinical response after 4-7 days of drainage via a catheter placed in the abscess cavity.

■ Multiple, large, or loculated abscess

■ Thick walled abscess with viscous pus

■ Concurrent intra-abdominal surgical pathology.

Bertel *et al *[24] compared open surgery with percutaneous drainage for the management of pyogenic liver abscesses greater than 5 cm. Whilst morbidity was comparable for both approaches, those who underwent open surgical resection/drainage required fewer secondary procedures, and achieved higher rates of abscess resolution. Very recently, it has been shown that using multi-detector CT to analyze abscesses could identify factors predictive of percutaneous drainage failure [25]. The strongest predictor of failure included the presence of gas in the abscess. Other notable factors were an abscess size greater than 7.3 cm, and a short length to the liver capsule of <0.25 cm. In the setting of failed percutaneous drainage of a liver abscess, another consideration prior to proceeding with open surgery is laparoscopic drainage. Aydin *et al *systematically reviewed the literature describing experience with this approach, and identified 53 cases of liver abscess treated by laparoscopy. The overall success rate was 90.5%, and no reported case required conversion to open [26]. From their review of this data, the authors concluded that laparoscopic surgery has the advantages of high success rates of open surgery, and the minimal invasiveness of percutaneous drainage. In light of this, it should be considered as an alternative before open drainage, when other modalities have failed.

### Actinomycotic liver abscess

Primary actinomycotic liver abscess is extremely rare. In this series, we include the first report to our knowledge of this type of liver abscess in Ireland and the United Kingdom. Actinomycosis is a chronic suppurative granulomatous disease characterized by abscess formation, tissue fibrosis and discharging sinuses. The actinomyces organism is a gram-positive, semi-facultative, pleomorphic non-spore-forming, non-acid-fast bacillus of the genus *Actinomyces*. The most common presentation of this disease is infection of the oral and cervicofacial region. However, the thoracic region, abdomino-pelvic region and CNS are also frequently involved. Although actinomyces species are part of the normal oral and gut flora, (where they have low pathogenicity) any disruption of the gastrointestinal mucosal barrier can lead to disease. Once the organism breaches mucosal barriers and is established locally, it is notoriously pathogenic and spread of infection has no regard for anatomic planes or boundaries. The few previously described cases of actinomycotic GI infection have occurred where there has been a loss of mucosal integrity, as occurs with surgery, appendicitis, diverticulitis, trauma, or foreign bodies including intrauterine contraceptive devices [27,28]. Possible causes of hepatic infection by actinomyces include disseminated infection from oral disease via the hepatic artery, from intra-abdominal disease via the portal vein, or from direct extension of a subdiaphragmatic or subhepatic process. However, in the majority of cases no cause is identifiable, as in our case. The 16 year old female in this series had no history of foreign travel, infectious contacts, past medical or surgical illness, abdominal trauma or foreign body in-situ. In addition, her dental hygiene was good, and she had no evidence of oral or cervical infection. Culture of the organism is difficult; it requires anaerobic or microaerophillic conditions, gram stain of the organism, haematoxylin-eosin stain of the 'sulfur granules', or more specific stains such as Grocott-Gomori methenamine-silver nitrate stain and P-aminosalicylic acid (Figure 2e &2f). Management of an actinomycotic liver abscess should follow the general principles described above, primarily with antimicrobial agents and abscess drainage. Actinomyces is sensitive to penicillin; the recommended course for hepatic actinomycosis is parenteral penicillin for 2-6 weeks, followed by oral therapy with penicillin or amoxicillin for 6-12 months [29]. Drainage of these liver abscesses is guided by their size, complexity and the clinical condition of the patient. Our patient required transfer to a specialized liver unit for surgical management because of clinical deterioration despite maximal non-operative therapy. Following hemi-hepatectomy and the recommended course of penicillin based systemic therapy she made an excellent recovery and remains well after 36 months.

## Conclusion

Although our series of hepatic abscess cases is small, there are few reports in recent years of outcomes with modern management techniques and thus our experience may provide valuable information to clinicians who encounter this uncommon condition. In particular, the rare case of an actinomycotic liver abscess in a healthy Irish female is interesting and noteworthy. Based on our review of existing literature and our experience to date, we propose the following summary to guide the management of pyogenic hepatic abscess:

1. Prompt administration of empiric broad spectrum parenteral antibiotics

2. Ultrasound and/or CT scan to confirm diagnosis, with simultaneous radiologically guided aspiration of all abscesses >3 cm, +/- drain placement.

3. Microbiological analysis of abscess aspirates and blood cultures: antibiotic regimen should be adjusted according to culture results and sensitivities.

4. Early recognition of septicaemia or organ failure and appropriate transfer to critical care unit. Consider repeat imaging to confirm correct drain placement and to determine response to treatment and final resolution of the abscess.

5. Surgical intervention should be considered for patients with large, complex, septated or multiple abscesses, underlying disease or in whom percutaneous drainage has failed.

## Consent

Written consent for publication was obtained from all patients whose clinical images are used in this article.

## Competing interests

The authors declare that they have no competing interests.

## Authors' contributions

HMH was involved in conception and design of the study, literature review, acquisition of data, analysis and interpretation of the data, and drafted the manuscript. STM was involved in data acquisition, drafting and editing the manuscript. NH contributed to data acquisition, literature review and manuscript preparation. RR was the radiologist involved in diagnostic imaging and radiologically guided management of all cases. NN was the pathologist involved in the surgical cases, made the rare diagnosis of Actinomycosis, acquired images for Figure 2, and edited the manuscript. OT and RW directed management of the patients clinically, performed the surgeries, supplied relevant clinical information about the patients and revised the manuscript for important intellectual content. All authors read and approved the final manuscript.
